# Retraction: Targeted non AR mediated smart delivery of abiraterone to the prostate cancer

**DOI:** 10.1371/journal.pone.0321800

**Published:** 2025-04-02

**Authors:** The PLOS One Editors

After this article [1] was published, concerns were raised regarding results presented in Figures 2, and 5-8.

Specifically:

- The following panels appear similar despite representing different experimental conditions:
  ◦ Figure 2c in [1] and Fig 2c in [2]
  ◦ Figure 5Bc in [1] and Figure 5Bj in [2]
  ◦ Figures 8Bd and 8Bt
- The following panels appear to partially overlap despite representing different experimental conditions:
  ◦ Figures 5Ac and 5Al
  ◦ Figure 5Ai in [1] and Figure 5Af in [2]
  ◦ Figures 6Ab and 6Ai
  ◦ Figures 7Ak and 7Ac
  ◦ Figure 8Al in [1] and Fig 8Ah in [2]
- There is substantial text overlap between the Results sections of [1] and [2].

The corresponding author stated that they disagreed with some of the concerns, but noted that errors were made during the preparation of the Figures 5B, 8A, and 8B. They provided replacement images for some affected panels, underlying image data for figures of concern, and quantitative data to support results reported in graphs in Figures 3 and 5-8. The data provided did not resolve most of the concerns noted above. In reviewing this matter, PLOS identified additional concerns about the data provided, as well as several other instances in which results reported in [2] were reused in [1].

Furthermore, the related article [2] was not declared when the *PLOS One* article was submitted, as is required by the PLOS policy on Submission and Publication of Related Studies, and PLOS did not receive documentation confirming that the authors had permission to republish the content from [2] in the *PLOS One* article.

In light of the above issues, the *PLOS One* Editors retract this article.

MSK did not agree with retraction. AB, MK, and IU either did not respond directly or could not be reached.

The retracted article [1] was removed from the *PLOS One* website at the time of retraction due to the similarities with [2]. The article’s Copyright and Data Availability statements were also updated at the time of retraction, and the removed contents are no longer offered under the Creative Commons Attribution License.
